# Clinical human metapneumovirus isolates show distinct pathogenesis and inflammatory profiles but similar CD8^+^ T cell impairment

**DOI:** 10.1128/msphere.00570-23

**Published:** 2024-01-10

**Authors:** Jorna Sojati, Yu Zhang, John V. Williams

**Affiliations:** 1 Department of Pediatrics, University of Pittsburgh School of Medicine, Pittsburgh, Pennsylvania, USA; 2 Program in Microbiology & Immunology, University of Pittsburgh School of Medicine, Pittsburgh, Pennsylvania, USA; 3 Department of Microbiology & Molecular Genetics, University of Pittsburgh School of Medicine, Pittsburgh, Pennsylvania, USA; 4 Institute for Infection, Immunity, and Inflammation in Children, Children’s Hospital of Pittsburgh, Pittsburgh, Pennsylvania, USA; Johns Hopkins University Bloomberg School of Public Health, Baltimore, Maryland, USA

**Keywords:** respiratory infection, human metapneumovirus, airway immunity

## Abstract

Human metapneumovirus (HMPV) is a negative-sense single-stranded RNA virus in the *Pneumoviridae* family and a leading cause of acute upper and lower respiratory infections, particularly in children, immunocompromised patients, and the elderly. Although nearly every person is infected with HMPV during early childhood, re-infections occur often, highlighting difficulty in building long-term immunity. Inflammatory responses, including PD-1-mediated impairment of virus-specific CD8^+^ T cells (T_CD8_), contribute to HMPV disease severity. HMPV strains are divided into four lineages: A1, A2, B1, and B2. However, little is known about immune responses to different viral subtypes. Here, we characterize responses to four HMPV clinical isolates—TN/94-344 (A1), TN/94-49 (A2), C2-202 (B1), and TN/96-35 (B2)—*in vivo* in C57BL/6 (B6) mice. TN/94-49 was avirulent, while TN/94-344, C2-202, and TN/96-35 showed varying degrees of weight loss and clinical disease. Differences in disease did not correlate to virus burden in upper or lower tracts. TN/94-49 HMPV exhibited highest nose titers and delayed lung clearance. Cytokine profiles differed between HMPV isolates, with TN/96-35 inducing the broadest lung inflammatory cytokines. TN/96-35 also showed lower HMPV burden and less weight loss than other virulent isolates, suggesting a more efficient antiviral response. Interestingly, disease correlated with higher expression of T-cell chemoattractant CXCL9. All isolates elicited PD-1 upregulation and decreased IFNγ and CD107a expression in virus-specific T_CD8_, with little difference between HMPV subtypes. This work uncovers previously uncharacterized variations in immune responses to clinical HMPV isolates of different lineages.

**IMPORTANCE:**

This study extensively explored differences in T-cell-mediated immunity between human metapneumovirus (HMPV) clinical isolates. Much existing HMPV research has been done with strains passaged extensively in cell lines, likely acquiring mutations advantageous to *in vitro* replication. Clinical isolates are collected directly from human patients and have undergone <10 passages, serving as more physiologically relevant models of HMPV infection. Additionally, existing animal studies of HMPV disease mainly focus on lung pathogenesis, while HMPV infects both upper and lower airways of humans. This work highlights distinct differences in HMPV burden in upper and lower tracts between clinical isolates. Lastly, this study uniquely explores differences in host immunity between all four HMPV genetic lineages. The predominant HMPV subtype in circulation varies seasonally; thus, understanding host responses to all subgroups is critical for developing effective HMPV vaccines.

## INTRODUCTION

Human metapneumovirus (HMPV), first described in 2001, is a primary cause of upper and lower airway infections and estimated to account for 4–16% of acute respiratory infections globally (1, 2). Although nearly all people acquire primary HMPV infection before age 5 years, re-infections occur often and highlight difficulty in building long-lasting immunity. Disease is more severe in children, immunocompromised patients, and older adults (3–6). Mild, self-limiting HMPV disease primarily involves upper respiratory tract symptoms including cough, coryza, and rhinorrhea, while more severe disease leading to hospitalizations is characterized by lower respiratory tract symptoms like bronchiolitis and pneumonia (7). There are two characterized genetic lineages of HMPV (classified as A and B), both further divided into two subgroups to create four distinct phylogenetic clades: A1, A2, B1, and B2 (8). However, differences in pathogenesis between subtypes remain poorly understood.

Like several other respiratory viruses, HMPV is distributed in a seasonal pattern, with the dominant genetic group in circulation varying from season to season. Thus, understanding differences in immune responses elicited by all four HMPV subgroups is important for optimizing HMPV vaccination. We sought to better characterize immune responses to all four lineages through representative clinical HMPV isolates—TN/94-344 (A1), TN/94-49 (A2), C2-202 (B1), and TN/96-35 (B2)—*in vivo* in C57BL/6 (B6) mice. Clinical isolates, which are virus strains isolated directly from patients and have undergone <10 passages *in vitro* in cells, have not experienced selective pressure to become laboratory-adapted strains. Thus, they serve as more physiologic representations of how virus replicates and induces immune activity in human airways.

## RESULTS

We first characterized isolate differences in HMPV-induced disease and pathogenesis induced using a permissive mouse model of upper and lower airway HMPV infections (9). We assessed weight loss and clinical disease scores of HMPV-infected mice as surrogate measures of disease severity. An equivalent inoculum of each strain was administered to mice intratracheally to induce mild-to-moderate disease as seen in most human infections.

**Fig 1:**
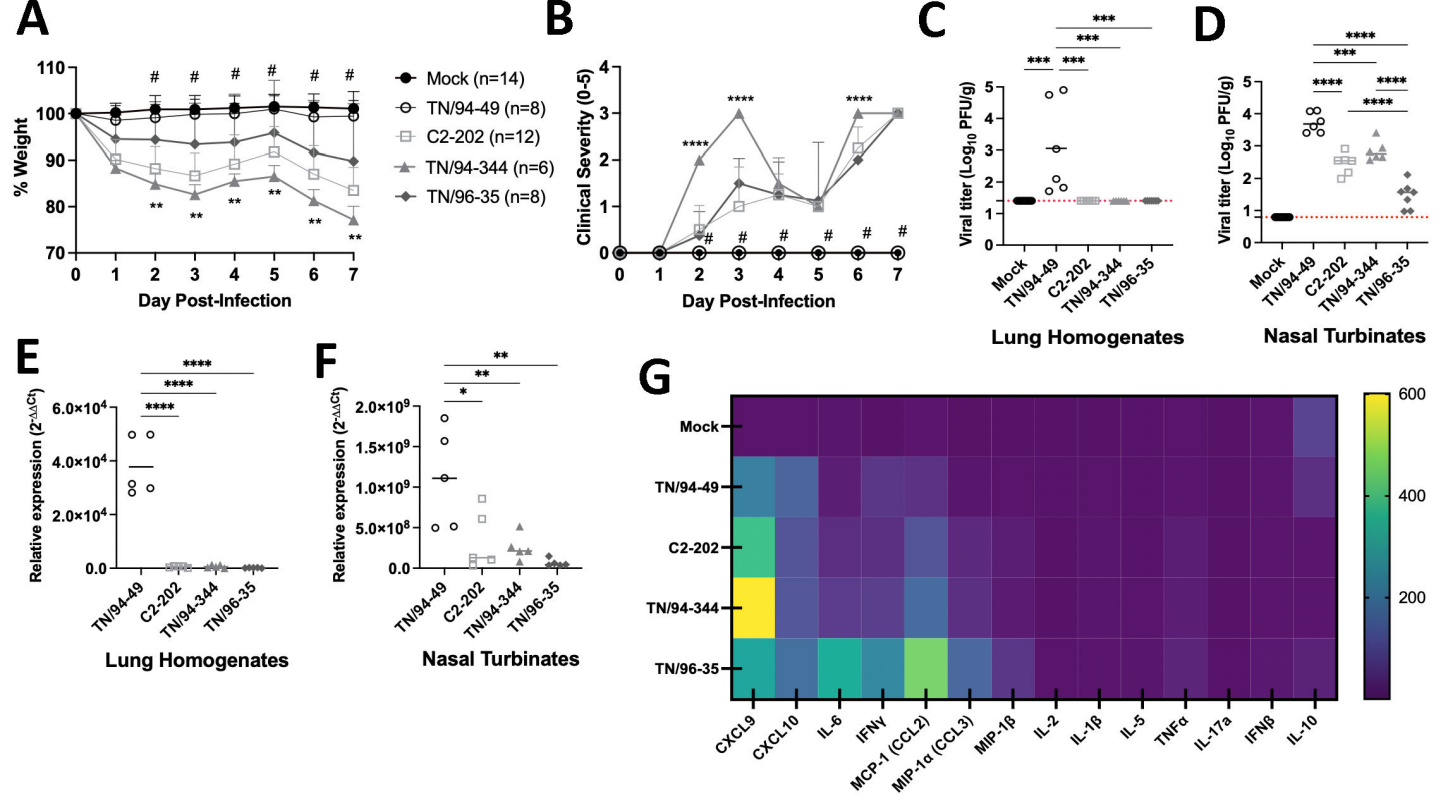
HMPV isolates vary in pathogenesis, airway burden, and lung cytokine profiles. (**A**) Body weight (% on day 0) of C57BL/6 (B6) mice infected with 5E5 plaque-forming units (PFU) or mock cell lysate of each HMPV strain. Two-way ANOVA, #*P* < 0.001 for TN/94-49 vs. all other clinical isolates, **P < 0.01 for TN/94-344 vs. TN/96-35. (**B**) Clinical disease score for mice described for panel A. One point is given for each of the following criteria: hunching, huddling, fur ruffling, rapid breathing, and lethargy. Two-way ANOVA, #*P* < 0.001 for TN/94-49 vs. all other clinical isolates. ****P < 0.0001 for TN/94-344 vs. all other clinical isolates. (**C and D**) Lung (**C**) and nasal turbinate (**D**) HMPV titers by plaque assay (plaque-forming units per gram) on day 7 post-infection of mice described for panel A. LOD noted by red dashed line. (**E and F**) Lung (**E**) and nasal turbinate (**F**) HMPV viral RNA detection by RT-qPCR on day 7 post-infection of mice described for panel A. Data are normalized to *Hprt* housekeeping gene and null condition of mock-infected mouse tissue by the 2^−∆∆Ct^ method. One-way ANOVA for (C-F), *P < 0.05, **P < 0.01, ****P < 0.0001. (**G**) Heat map representing protein expression levels (ng/mL) of inflammatory cytokines in lung homogenates collected on day 7 post-infection from mice described for panel A.

Isolate TN/94-49 (A2) was avirulent in mice, showing no weight loss or physical measure of disease (Fig. 1A and B). Of the three disease-causing strains, TN/96-35 (B2) caused milder weight loss (~10%) than C2-202 (B1) infection (~15%) but similar disease scores. TN/94-344 (A1) caused greater weight loss (>20%) and higher clinical disease scores early post-infection. We then assessed whether disease correlated to HMPV burden in upper and lower airways by plaque assay (Fig. 1C and D) and RT-qPCR (Fig. 1E and F). Of note, all three disease-causing HMPV strains showed minimal detectable viral RNA and no infectious plaque formation, suggesting virus clearance, from the lung by day 7 post-infection, while the avirulent TN/94-49 strain persisted in the lung at a high viral load (Fig. 1C and E).^10^ ^11^Virus was still detected in the upper airways of all four HMPV strains (Fig. 1D and F). Avirulent TN/94-49 was also detected at the highest titer in nasal tissue. Of the three virulent strains, C2-202 (causing moderate disease) and TN/94-344 (causing more severe disease) showed similar nasal HMPV burden. TN/96-35, which showed the least weight loss of all disease-causing strains, was also detected in lowest titers in upper airways. We measured lung inflammatory cytokines by Luminex assay and saw that TN/96-35 upregulated several cytokines from the panel, including molecules that drive T-cell chemotaxis (CXCL9 and CXCL10), macrophage recruitment (MCP-1 and MIP-1α) and activation (IFNγ), and systemic inflammation (IL-6) (Fig. 1G). While HMPV disease did not correlate with HMPV burden, it correlated with expression levels of CXCL9, with TN/94-49 lowest and TN/94-344 highest expressions.

Next, we compared differences in CD8^+^ T cells (T_CD8_) among clinical isolates, which play a major role in facilitating HMPV clearance (12–14). HMPV infection leads not only to recruitment of T_CD8_ to the lung but also to induction of virus-specific T_CD8_ that becomes acutely impaired in the response to infection (15). Key hallmarks of acute T_CD8_ impairment are reduced secretion of cytotoxic molecules and increased expression of inhibitory receptors, namely, PD-1 (16, 17).

**Fig 2:**
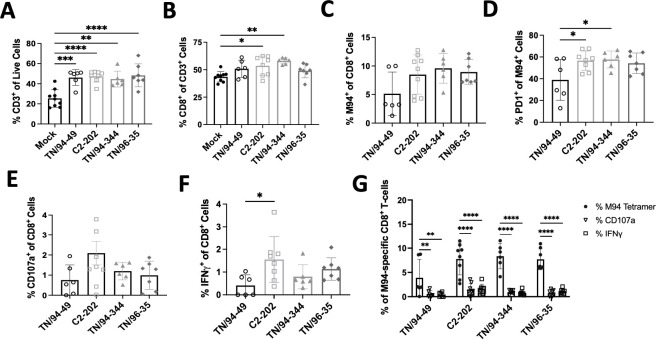
HMPV isolates induce dysfunctional T_CD8_ with high PD-1 expression and reduced effector function. (**A**) Frequency of total CD3^+^ lymphocytes (% of total live cells) in lungs of mice infected with 5E5 PFU HMPV and euthanized day 7 post-infection. (**B–D**) Frequency of total lung CD8^+^ lymphocytes (% of total CD3) (**B**), virus-specific (M94^+^) T_CD8_ lymphocytes (% of total CD8) (**C**), and PD-1^+^ HMPV-specific T_CD8_ (% of total M94^+^ T_CD8_) (**D**) in lungs of mice described for panel A. (**E and F**) Frequency of lung T_CD8_ expressing CD107a (**E**) or IFNγ (**F**) after 5 hours of *ex vivo* stimulation with HMPV M94 peptide. One-way ANOVA for panels A–F, *P < 0.05, *P < 0.05, **P < 0.01, ****P < 0.0001. (**G**) Representation of functionality for M94-responsive lung T_CD8_ cells showing % M94^+^ cells and % CD107a/IFNγ production in response to M94 peptide stimulation. Two-way ANOVA, **P < 0.01, ****P < 0.0001.

**Fig 3:**
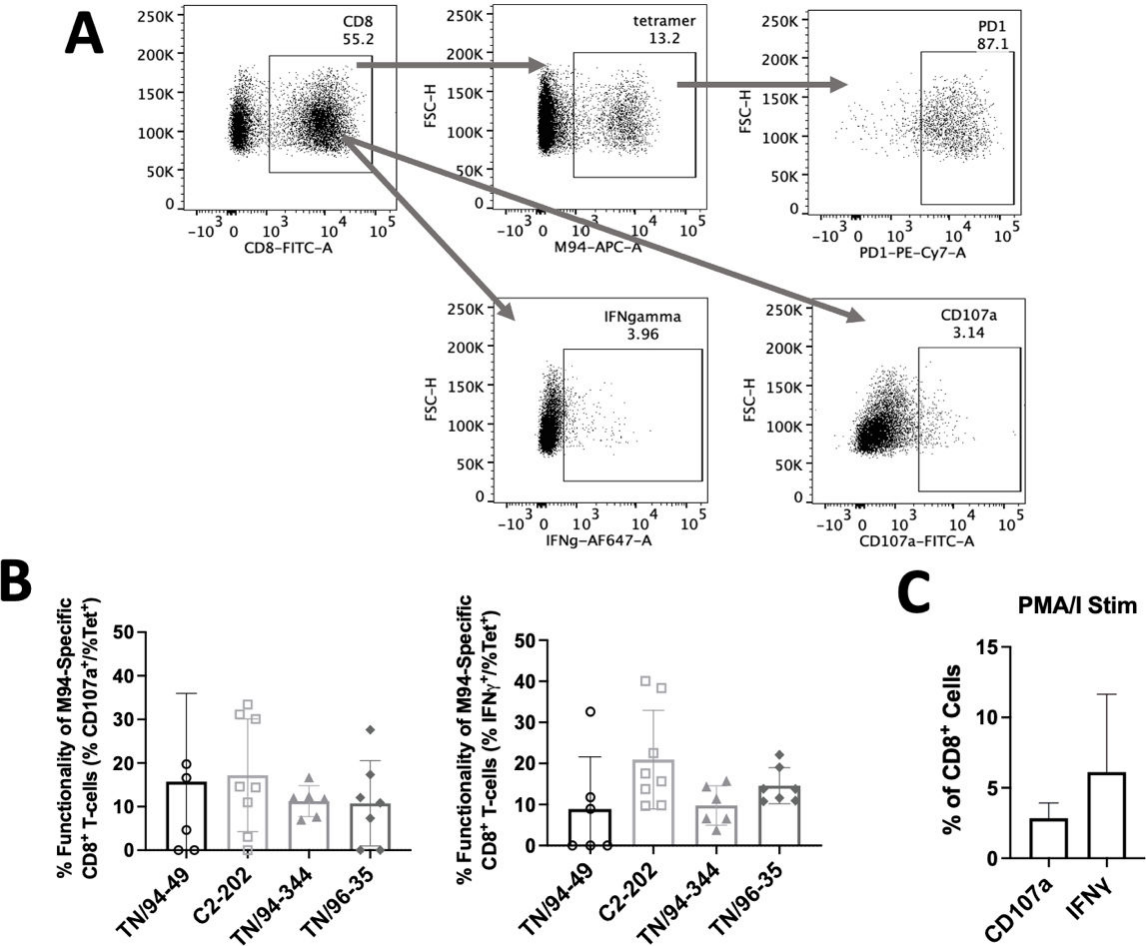
Characterization of virus-specific T_CD8_ impairment during HMPV infection. (**A**) Gating strategy for tetramer^+^, PD-1^+^, IFNγ^+^, and CD107a^+^ T_CD8_ cells. (**B**) Functionality plots for CD107a and IFNγ in M94-specific cells, plotted as % CD107a^+^ or IFNγ^+^ on M94 stimulation divided by % M94^+^ cells by tetramer staining, for mice described in Fig. 2A. (**C**) Positive control of lung T_CD8_ stimulation using PMA/ionomycin for mice described in Fig. 2A.

**Fig 4:**
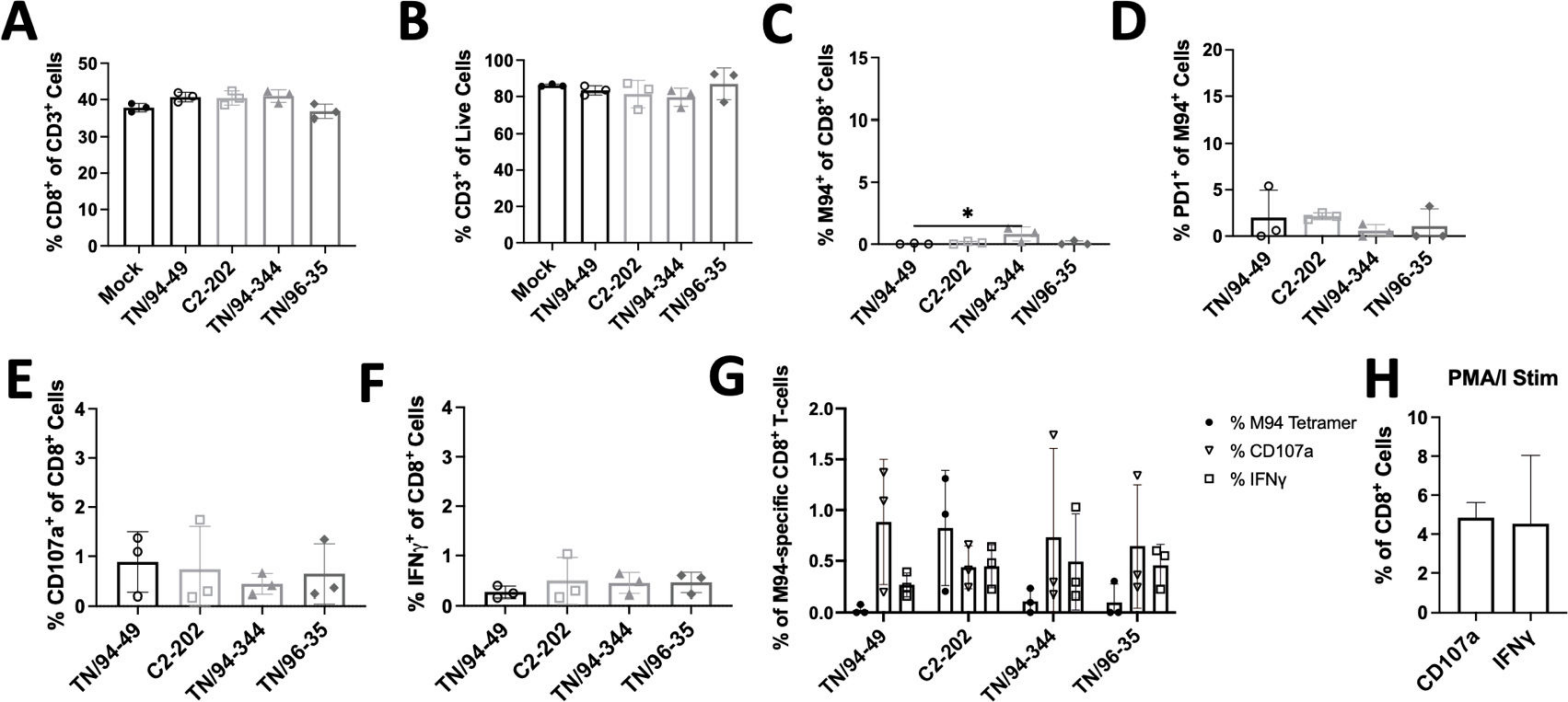
Virus-specific T_CD8_ are minimally detected in spleen tissue. (**A–D**) Frequency of total CD3^+^ lymphocytes (% of total live cells) (**A**), total lung CD8^+^ lymphocytes (% of total CD3) (**B**), virus-specific (M94^+^) T_CD8_ lymphocytes (% of total CD8) (**C**), and PD-1^+^ HMPV-specific T_CD8_ (% of total M94^+^ T_CD8_) (**D**) in spleens of mice described in Fig. 2A. (**E and F**) Frequency of spleen T_CD8_ expressing CD107a (**E**) or IFNγ (**F**) after 5 hours of *ex vivo* stimulation with HMPV M94 peptide. (**G**) Functionality of M94-responsive spleen T_CD8_ cells showing % M94^+^ cells and % CD107a/IFNγ production in response to M94 peptide stimulation. (**H**) Positive control of spleen T_CD8_ stimulation using PMA/ionomycin.

Infection with all four HMPV isolates led to recruitment of CD3^+^ T cells to the lung (Fig. 2A), with C2-202 and TN/94-344 (the two strains showing greatest weight loss) in particular showing increased lung T_CD8_ frequency (Fig. 2B). There was no difference in induction of HMPV-specific T_CD8_ using an MHC-I tetramer bound to an immunodominant HMPV epitope M_94-102_ (called M94) between isolates, but the three virulent strains notably exhibited greater PD-1 upregulation on virus-specific T_CD8_ than avirulent TN/94-49 (Fig. 2C and D). Nonetheless, all four isolates showed abundant PD-1 expression. To explore whether these PD-1 expressing T_CD8_ cells were impaired, we assessed functionality by *ex vivo* stimulation with M94 HMPV peptide and measuring response by CD107a (degranulation marker) and IFNγ expression. Only 1%–2% of T_CD8_ cells were functional (CD107a+ or IFNγ+) in response to M94 peptide across all HMPV isolates (Fig. 2E and F), while 5%–10% of lung T_CD8_ were M94 specific by tetramer staining, highlighting markedly reduced function and T_CD8_ impairment across all four genetic clades (Fig. 2G and 3) Importantly, induction of dysfunctional virus-specific T_CD8_ was largely lung specific, with very few tetramer specific, PD-1-expressing, or HMPV peptide-responsive T_CD8_ detected in the spleen (Fig. 4).

## DISCUSSION

Here, we highlight notable differences in clinical disease caused by isolates spanning all four HMPV lineages. Isolate TN/96-35 (B2) and C2-202 (B1) both induced mild HMPV disease. There was a starker contrast between members of the A genotype: TN/94-344 (A1) demonstrated highest weight loss and most severe infection of isolates tested, while TN/94-49 (A2) was avirulent but showed highest nasal burden and delayed lung clearance. HMPV pathogenesis varied significantly among the four phylogenetically distinct isolates, and differences in disease did not correlate to HMPV burden.

Existing research of clinical disease between HMPV subgroups demonstrate mixed results. Two studies of HMPV-infected patients showed that pneumonia is more common and disease severity is higher with HMPV A infection (18, 19), and another showed that genotype A-infected patients have higher nasopharyngeal viral load (20). In contrast, a study of hospitalized HMPV-infected children showed higher severity with genotype B infection (21), and another report demonstrated higher frequency of laryngitis symptoms in genotype B-infected patients (22). Other groups show no difference in clinical presentation by virus genotype (23, 24).

While there may clearly be a lack of consensus in standardizing disease severity among the mentioned studies, another pertinent confounder could be clinical differences within A and B subgroups as demonstrated in our study.

Importantly, we found distinct lung inflammatory cytokine profiles among HMPV isolates. TN/94-344 infection (which showed lowest nasal HMPV titer and most weight loss) induced the broadest degree of cytokines. One study to date has also explored cytokine profiles between HMPV subtypes, which showed no differences at the protein level (25). Interestingly, we saw a clear correlation between HMPV disease and levels of T-cell chemoattractant CXCL9. Several groups, including ours, have demonstrated the importance of T cells and specifically T_CD8_ in mediating HMPV airway disease (13) and facilitating HMPV clearance (12, 14). We previously described a PD-1-driven acute impairment phenotype in A2 HMPV (16, 17).

Here, we demonstrate a similar T_CD8_ impairment phenotype among isolates of all four HMPV clades. All strains elicited virus-specific lung T_CD8_ with PD-1 upregulation and reduced CD107a and IFNγ production upon peptide stimulation. We show previously with A2 HMPV that T_CD8_ impairment can be abrogated by PD-1 inhibition (16). This work provides support for exploring potential PD-1 blockade therapy in severe infection among all HMPV subtypes.

We reveal clear differences in virulence and cytokine induction among four HMPV isolates and highlight the necessity of characterizing phenotypes for all HMPV genetic groups in future research and clinical studies.

## SUPPLEMENTAL MATERIAL

Supplemental material msphere.00570-23-s0001.docx Supplemental methods.

ASM does not own the copyrights to Supplemental Material that may be linked to, or accessed through, an article. The authors have granted ASM a non-exclusive, world-wide license to publish the Supplemental Material files. Please contact the corresponding author directly for reuse.
