# Supplementary material for: Clinical human metapneumovirus isolates show distinct pathogenesis and inflammatory profiles but similar CD8+ T cell impairment
Source: mSphere. 2024 Jan 10;9(1):e00570-23. doi: 10.1128/msphere.00570-23 (PMC10826344; doi:10.1128/msphere.00570-23)
Supplement: Supplemental material — Supplemental methods. [file msphere.00570-23-s0001.docx]

**Supplementary Methods:**

**HMPV Isolate Preparation:** All virus strains used were obtained from nasopharyngeal washes of patients with upper or lower respiratory tract illness as described previously (24). HMPV isolates TN/94-344 (A1), TN/94-49 (A2), C2-202 (B1), and TN/96-35 (B2) were grown in LLC-MK2 cells under enriched media conditions and sucrose-purified as described (24). Purified virus stock was titrated by plaque assay in LLC-MK2 cell monolayers and virus was quantified by plaque-forming units/mL (25). Virus stocks used in animal studies had all undergone <10 passages in LLC-MK2 cells.

**Animals & Infections:** Six-to-eight-week-old C57BL/6 (B6) mice were purchased from Jackson Laboratory. All animals were maintained in specific pathogen-free conditions in accordance with University of Pittsburgh Institutional Animal Care and Use Committee (IACUC) guidelines. For experiments, mice were anesthetized by inhaled isoflurane (5% isoflurane in 100% O_2_, flow rate 2.5 L/min) and infected intratracheally (I.T.) with 5x10^5^ total plaque-forming units of HMPV.

**Virus Plaque Titration & Cytokine Quantitation:** Virus quantitation was done by plaque titration of clarified homogenates from lung and nasal turbinate tissue as previously described (25). Multiplex immunoassay (ProcartaPlex, Thermo Fisher) of undiluted lung homogenate was performed to determine inflammatory cytokine levels according to manufacturer’s instructions.

**qPCR**: RNA was extracted from 100-uL volume of lung or nasal turbinate homogenate using RNeasy kit (Qiagen) according to manufacturer’s instructions. Quantitative reverse-transcription PCR (RT-qPCR) was performed by preparing 25-$\mu$L reaction volume mixture containing 5-$\mu$L extracted RNA using AgPath-ID One-Step RT-PCR Reagents (Applied Biosystems). TaqMan primers and probes were used according to manufacturer’s instructions (Applied Biosystems). HMPV was detected using primers and probes targeting the N gene as previously published (26). Values were normalized to the housekeeping gene *Hprt* and to mice receiving mock infection with cell lysate using the 2-∆∆Ct method.

**Tetramer Staining:** Flow cytometric staining of lymphocytes isolated from mouse lung and spleen was done as described (14). Lung and spleen tissue were minced, digested enzymatically with 2 mg/mL collagenase A and 20 mg/mL DNase (Roche) for 60 minutes at 37^0^C, and passed through a 70 $\mu$M cell strainer to obtain single cell suspensions. Cells were next incubated with red blood cell lysis buffer (Sigma-Aldrich). 1x10^6^ lymphocytes per sample were stained with: LIVE/DEAD violet dye (Thermo-Fisher), anti-CD8a (clone 53–6.7, BD Biosciences), anti-CD3 (clone 145-2C1, BD Biosciences) anti-CD19 (clone 1D3, eBioscience), and anti-PD-1 (clone J43, BD Biosciences). Cells were also incubated with an APC-labeled tetramer generated against HMPV epitope H2-K^b^/M_94–102_ (VALDEYSKL) (M94) to stain HMPV-specific cells. All surface staining was done for 60 minutes at room temperature. Background staining was determined by staining with a non-specific influenza tetramer. All data were collected using the BD LSR II flow cytometer (BD Biosciences) and analyzed using FlowJo software (Tree Star).

**Peptide stimulation & ICS:** In parallel with tetramer staining, 1x10^6^ lymphocytes from the same mouse sample were restimulated *in vitro* using M94 peptide in the presence of anti-CD107a (clone 1D4B, BD Biosciences), brefeldin A, and monensin (BD Biosciences) for 5 hours as described (14). PMA/Ionomycin (50 ng/mL PMA, 2 $\mu$g/mL ionomycin) stimulation served as a positive control. Following surface staining for CD3, CD8, and CD19 as above, cells underwent fixation/permeabilization and intracellular cytokine staining (ICS) was performed using anti-IFN$\gamma$ (clone XMG1.2, BD Biosciences). Background staining was determined for CD107a and IFN$\gamma$ by stimulation with an irrelevant influenza peptide. Data were collected using the BD LSR II flow cytometer (BD Biosciences) and analyzed using FlowJo software (Tree Star).

**Statistical Analyses:** Data analysis was performed using Prism version 9.0 (GraphPad software). Multiple group comparisons were done for all figures using either one-way (if one independent variable) or two-way (if two or more independent variables) ANOVA. Data are represented as mean + standard deviation.
